# Altered Mental Status on Top of Anaplasmosis-Induced Severe Rhabdomyolysis: A Rare Clinical Presentation

**DOI:** 10.7759/cureus.45020

**Published:** 2023-09-11

**Authors:** Aurangzeb Memon, Abdelmalek Abdelghany, Mohammed Abusuliman, Mohamed Eldesouki, Minahil Fatima, Omar Abdelhalim, Hazem Abosheaishaa

**Affiliations:** 1 Internal Medicine, Icahn School of Medicine at Mount Sinai, Queens Hospital Center, New York, USA; 2 Internal Medicine, Faculty of Medicine, October 6 University, Cairo, EGY; 3 Internal Medicine, Henry Ford Health System, Detroit, USA; 4 Internal Medicine, Mayo Clinic, Rochester, USA; 5 Internal Medicine, Services Hospital Lahore, Lahore, PAK; 6 Internal Medicine, Icahn School of Medicine at Mount Sinai, New York City (NYC) Health and Hospitals, Queens Hospital Center, New York, USA; 7 Internal Medicine/Gastroenterology, Cairo University, Cairo, EGY

**Keywords:** fever, aki, ams, rhabdomyolysis, anaplasmosis

## Abstract

Human granulocytic anaplasmosis (HGA) is a disease caused by tick-borne infection of Anaplasma phagocytophilum. The typical symptoms are fever, malaise, and body aches accompanied by abnormal blood tests such as leukopenia, thrombocytopenia, and transaminitis. Some rare complications may occur, especially in patients living in heavily wooded areas, with a mean age of 70 years. We present a case of a 67-year-old male who was admitted for lower abdominal pain, fever, and diarrhea with derangement of his blood tests. Despite treatment, his condition deteriorated and complicated rhabdomyolysis and acute kidney dysfunction. Empiric treatment including doxycycline was initiated while waiting for the infection blood work results. PCR came back positive for HGA. Empiric therapy was narrowed down to doxycycline for 14 days, and the patient's condition began to improve gradually and steadily. Aggressive hydration markedly improved rhabdomyolysis and, in turn, kidney function. Our case underscores the importance of considering HGA in ambiguous clinical scenarios and highlights the value of early diagnosis, empiric treatment, and intravenous hydration, especially in the presence of rhabdomyolysis.

## Introduction

Human granulocytic anaplasmosis (HGA) is a tick-borne zoonotic disease caused by the rickettsial bacterium Anaplasma phagocytophilum [1]. A. phagocytophilum is an obligate, Gram-negative intracellular bacterium that is unique in its affinity to neutrophils. In sheep and cattle, it causes anaplasmosis, often known as tick-borne fever and pasture fever. A. phagocytophilum is genetically related to rickettsia and can be transmitted by the Ixodes scapularis tick in the northeast United States and Ixodes pacificus in California. After the tick bite transmits the bacterium, it disseminates to the bone marrow and spleen. The bacterium can only survive and grow in the cytoplasmic vacuoles and early endosome of the polymorphonuclear cells where it obtains nutrients for binary fission and forms tiny clusters known as morulae; it affects cells from the myeloid and granulocytic lineages [2]. A. phagocytophilum prevalence varies by area and is affected by tick populations and environmental variables [2]. The National Notifiable Diseases Surveillance System data from 2012 to 2016 showed 7.27 cases per million people per year with the numbers rising each year [3].

## Case presentation

A 67-year-old male, with no known past medical history, came to the ED with a five-day history of lower abdominal pain that started while he was pushing a cart in his garden. It was constant in nature, 6-10 in intensity, burning in nature, radiating to the upper abdomen, and associated with subjective fevers, nausea, food intolerance, and diarrhea. The patient mentioned that he had recently visited another hospital with the same complaint, where he was reassured after getting a CT scan done and sent home. He denied alcohol use, smoking, or illicit drugs. In the ED, vitals were as follows: temperature: 102.6 F; heart rate: 90 b/minute; blood pressure: 116/80; and oxygen saturation: 97%. Physical examination was grossly normal. In the ED, pan cultures were collected, and initial labs were done and revealed positive urine analysis (UA) with large blood, bacteria, and nitrites although with trace leukocyte esterase and normal WBCs, elevated kidney function along with high CRP, LDH, and prolactin (Table 1).

**Table 1:** Initial labs in the ED.

Component	Value	Reference range
CBC		
PLT	59	150-450 x10^3^/mcL
WBC	5.47	4.80-10.80 x10^3^/mcL
RBC	4.96	4.70-6.10 x10^6^/mcL
HGB	13.4	14.0-18.0 g/dL
HCT	39.2	42.0-52.0%
MCV	79.0	80.0-99.0 fL
MCV	27.0	27.0-31.0 pg
MCHC	34.2	29.8-35.2 g/dL
MPV	11.5	8.7-12.9 fL
RDW	13.1	12.0-15.0%
NRBC abs	0.00	≤0.00 x10^3^/mcL
NRBC %	0.0	0.0-0.0%
Neutrophil %	88.5	44.0-70.0%
Lymphocyte %	6.8	20.0-45.0%
Monocyte %	3.8	2.0-10.0%
Eosinophil %	0.0	1.0-4.0%
Basophil %	0.4	0.0-2.0%
Imm gran %	0.5	0.0-2.0%
Neutrophil abs	4.84	2.10-7.60x10^3^/mcL
Lymphocyte abs	0.37	1.00-4.90x10^3^/mcL
Monocyte abs	0.21	0.10-1.10x10^3^/mcL
Eosinophil abs	0.00	0.10-0.40x10^3^/mcL
Basophil abs	0.02	0.00-0.20x10^3^/mcL
Immature gran abs	0.03	0.00-0.20x10^3^/mcL
Reticulocyte %		0.50-1.50%
Reticulocyte abs		0.0221-0.0963x10^6^/mcL
Serum		
Sodium	137	136-145 mmol/L
Potassium	3.4	3.5-5.1 mmoL/L
Chloride	102	98-108 mmol/L
CO_2_	21	22-29 mmol/L
Glucose	140	74-110 mg/dL
Calcium	8.4	8.6-10.3 mg/dL
KFTs		
BUN	27	6-23 mg/dL
Creatinine	1.68	0.70-1.20 mg/dL
Anion gap	14	8-16 mEq/L
eGFR(cr)	44	≥60 ml/min/1.73 m^2^
LFTs		
Albumin	4.0	3.5-5.2 g/dL
Total protein	7.4	6.6-8.7 g/dL
Total bilirubin	1.30	0.00-1.20 mg/dL
Direct bilirubin	0.8	0.0-0.3 mg/dL
ALK PHOS	58	40-129 U/L
ALT (SGPT)	78	0-41 U/L
AST (SGOT)	241	5-40 U/L
Fibrinogen	414	200-393 mg/dL
Haptoglobin	217	
Procalcitonin (PCT)	15.51	0.02-0.10 ng/mL
Highly sensitive C-reactive protein	292.50	≤5.00 mg/L
LDH	572	50-242 U/L

ECG was insignificant for any acute changes. Chest x-ray was unremarkable, and CT during arterial orthography (CTAP) showed multiple low-density nodules throughout both hepatic lobes, which likely represented cysts. No solid mass.

Given the positivity of the UA, the patient was started on ceftriaxone 1 g IV daily for two days; however, he continued to spike fever up to 102 F, and his consciousness level deteriorated, with difficulty speaking in clear sentences and multiple pauses with a trembling voice, so antibiotics changed to vancomycin and Zosyn due to the unclear nature of the infection and impending sepsis. The patient started to deny treatment and started to lose understanding of his condition and the treatment, which was being provided. On the third day of admission, metronidazole was added to the regimen to have anti-parasitic coverage. Further collateral information was provided by the patient’s family, which revealed that the patient was a naturopathic doctor who traveled all over the world to cure diseases with natural substances and had recent visits to South Africa and Texas in the previous six weeks.

Subsequently, metronidazole and Zosyn were discontinued, and the patient was started on acyclovir, meropenem, and doxycycline, along with continuing vancomycin as per the infectious diseases’ recommendations. Blood samples were sent for parasite smear, malaria PCR, babesia PCR, and serology, Ehrlichia/Anaplasma PCR, Ehrlichia serology, leptospirosis serology, QuantiFERON, and repeated blood cultures. A spinal tap was also ordered to rule out encephalitis/meningitis, HSV, VZV, and fungal/TB/syphilis etiology; however, the initial blood and urine cultures came back negative.

On the sixth day since admission, the patient’s mental status continued to worsen along with worsening kidney and liver functions (Table 2). New complementary labs were ordered (Table 2) and returned positive for rhabdomyolysis (CK: 12917) and negative for HIV, HAV, HBV, and ADAMTS13. The patient was still having a spiking fever and then started to mount the WBC response. The patient was put on hydration at 100 ml/hr to treat rhabdomyolysis.

**Table 2:** Follow-up and complementary labs on the sixth day.

Component	Value	Reference range
PLT	93	150-450x10(3)/mcL
WBC	11.38	4.80-10.80x10(3)/mcL
RBC	3.92	4.70-6.10x10(6)/mcL
HGB	10.5	14.0-18.0 g/dL
HCT	30.1	42.0-52.0%
MCV	76.8	80.0-99.0 fL
MCH	26.8	27.0-31.0 pg
CPK	12,917	20-190 U/L
Sodium	140	136-145 mmol/L
Potassium	3.6	3.5-5.1 mmoL/L
Chloride	110	98-108 mmol/L
CO2	19	22-29 mmol/L
BUN	52	6-23 mg/dL
Creatinine	3.54	0.70-1.20 mg/dL
Glucose	145	74-110 mg/dL
Calcium	7.4	8.6-10.3 mg/dL
Anion Gap	11	8-16 mEq/L
eGFR(cr)	18	≥60 ml/min/1.73 m^2^
Albumin	2.4	3.5-5.2 g/dL
Total protein	5.2	6.6-8.7 g/dL
Total bilirubin	2.20	0.00-1.20 mg/dL
Direct bilirubin	1.8	0.0-0.3 mg/dL
ALK PHOS	66	40-129 U/L
ALT (SGPT)	85	0-41 U/L
AST (SGOT)	281	5-40 U/L
ADAMTS13 activity	43.1	>66.8%

On the seventh day, patients’ kidney function slowly but steadily started to get better along with improved mental status. The infectious workup came back positive for Anaplasma PCR, and everything else was negative. All the other antibiotics were discontinued, except 14 days of doxycycline. Kidney functions came back to baseline on the 12th day (Table 3) after good hydration and treatment of anaplasmosis and rhabdomyolysis.

**Table 3:** Final labs on the 12th day.

Component	Value	Reference range
Sodium	137	136-145 mmol/L
Potassium	3.9	3.5-5.1 mmoL/L
Chloride	105	98-108 mmol/L
CO2	26	22-29 mmol/L
BUN	24	6-23 mg/dL
Creatinine	1.73	0.70-1.20 mg/dL
Glucose	95	74-110 mg/dL
Calcium	7.5	8.6-10.3 mg/dL
Anion gap	6	8-16 mEq/L

## Discussion

Anaplasmosis was first reported in the United States by Chen et al. [3] In 1994. Since then, the CDC has reported a steady increase in the incidence of the infection [4]. After ixodid ticks infect granulocytes, an acute febrile illness develops. The incubation period ranges from seven to 10 days, after which the clinical manifestations appear. High fever, chills, myalgia, arthralgia, headache, nausea, and dry cough are some of the most common symptoms of the infection [5]. In the majority of cases, patients are initially thought to have a minor viral infection, which usually clears up quickly. However, up to 3% may develop life-threatening complications, with a mortality rate of 1% [1].

Life-threatening complications of anaplasmosis include multiple organ failure and rhabdomyolysis. Rhabdomyolysis is a well-established and potentially fatal side effect of anaplasmosis infection, and it has been reported in multiple case reports [6-9,10-12]. The mechanism by which anaplasmosis specifically causes rhabdomyolysis is still unknown. However, Infection-related rhabdomyolysis is mediated by endotoxic effects, hypoxia, and up- or down-regulated enzymatic responses [13]. The adherence of neutrophils to endothelial cells is a key mechanism in tissue inflammation. Instead, A. phagocytophilum exhibits lower adhesion to endothelial cell lines and enhances cytokine release and macrophage activation [14].

Typical laboratory findings in anaplasmosis infection include leukopenia, thrombocytopenia, and elevated liver enzymes. A peripheral blood smear can detect the morula in the cytoplasm of infected circulating granulocytes. To confirm the diagnosis, serological assays such as indirect immunofluorescence antibodies and real-time PCR are usually used [15].

Rhabdomyolysis is a complex and serious disorder characterized by rapid disintegration of injured or damaged skeletal muscles. When myocytes’ integrity is compromised, intracellular muscle components, such as myoglobin, creatine kinase (CK), aldolase, lactate dehydrogenase, and electrolytes, are released into the systemic circulation and extracellular space. These components put kidney health at risk for nephrotoxicity or even renal failure in 33% of cases. Rhabdomyolysis can range from a symptomless syndrome with an elevated CK level to a potentially fatal condition with extremely high CK levels, electrolyte abnormalities, acute renal failure (ARF), and disseminated intravascular coagulation [16]. Despite being the most common cause of rhabdomyolysis, traumatic muscle injury is not the only etiology as it can also be caused by drugs, toxins, muscle ischemia, genetic disorders, and, as in our case, infection [17]. Rhabdomyolysis is clinically manifested by myalgia, limb weakness and swelling, gross proteinuria without hematuria [18], and lab values indicating leukopenia and thrombocytopenia. In the presented case, the history revealed Anaplasma infection, which was the most probable etiology for the presentation. Anaplasma infection has been reported on rare occasions to be associated with rhabdomyolysis and in a diversity of countries and continents [19].

Boateng et al. [6] reported a case of a woman, 69 years of age, residing in a northeastern US suburb tick-infested area, who presented with fever lethargy, and diarrhea. Initial blood studies detected, leukopenia, severe thrombocytopenia, high hepatic transaminase, and creatinine levels. A peripheral blood smear revealed typical morulae in the neutrophils which is a rapid diagnostic clue for human granulocytic anaplasmosis [20]. She was treated with normal saline for hydration and doxycycline as an antibacterial agent. Complete recovery was reported three months later. Another case was reported by Talsness et al. [7] in northern Wisconsin of an 84-year-old man, presenting with lethargy, fever, confusion, and urinary incontinence. Initial diagnostic tests, including x-ray blood culture and urine culture, were unfruitful and were initially treated with ceftriaxone assuming the diagnosis of meningitis. After further interviewing, a five-day-old tick bite in the patient’s groin was revealed. Further lab studies were conducted revealing neutrophil cytoplasmic inclusion typically seen with Anaplasma morulae. Administration of doxycycline, as well as hydration with sodium bicarbonate, was sufficient to improve the mental status and renal perfusion. An additional case study was conducted by Jawanda [9] in Connecticut of a Ghanaian female, 37-year-old, patient residing in Connecticut for more than a year. She had a typical presentation of infection-caused rhabdomyolysis including lethargy, myalgia, fever, and diarrhea. Her lab values indicated markedly elevated CK at 150,000 U/L, aspartate aminotransferase of 404 U/L, and alanine aminotransferase of 120 U/L. Her further workup tested her for multiple infections, which all turned out to be negative, including ehrlichiosis. However, she was presumed to have a tick-borne infection and was treated with doxycycline and aggressive IV fluids, which were sufficient for her recovery after 15 days. Cho et al. [12] presented a case in the Republic of Korea of a female patient, 84 years old. Her first symptoms were fever and dizziness, in addition to having a history of hypertension. Her lab workup revealed elevated cardiac enzymes, creatinine, BUN, and CRP. It also showed thrombocytopenia and a severely decreased GFR of 19.1. Peripheral blood smear was examined affirming the presence of morula inclusions in neutrophils suggestive of HGA. Treatment also consisted of doxycycline as well as ceftriaxone, leading to complete recovery and discharge after 12 days.

The challenge with HGA is that its clinical signs are ambiguous, frequently mimicking a viral infection. Early suspicion and diagnosis are especially critical when the infection is accompanied by rhabdomyolysis. Even if the history of a tick bite is not affirmative, the blood film examination is negative, or while waiting for the results of specific A. phagocytophilum diagnostic tests such as paired serology or PCR on acute phase blood, a history of a tick bite and a high level of clinical suspicion is enough to warrant consideration for doxycycline treatment [1]. Aggressive fluid resuscitation and electrolyte replenishment are the mainstay of rhabdomyolysis treatment and are essential to avoid pre-renal azotemia and renal failure [21].

## Conclusions

Anaplasmosis is an emerging infectious illness that can cause rhabdomyolysis, acute kidney injury, and altered mental status, especially in old age. This example underscores the necessity of suspecting tick-borne infections as anaplasmosis in feverish, multisystem-affected patients, especially with travel history and when traditional diagnostic testing is inconclusive.
